# Comparison of Atezolizumab plus Aevacizumab and Atezolizumab plus Aabozantinib for advanced hepatocellular carcinoma: A cost-effectiveness analysis

**DOI:** 10.1371/journal.pone.0337606

**Published:** 2025-12-03

**Authors:** Heng Xiang, Zhihua She, Liting Wang, Ye Peng, Lei Zhang, Chongqing Tan

**Affiliations:** 1 Department of Pharmacy, the Second Xiangya Hospital of Central South University, Changsha, Hunan, China; 2 Department of Pharmacy, the Hunan Provincial Maternal and Child Health Care Hospital, Changsha, Hunan, China; 3 Department of Nephrology, the Second Xiangya Hospital of Central South University, Changsha, Hunan, China; PearlsInMires, KOREA, REPUBLIC OF

## Abstract

**Background:**

Atezolizumab combined with either bevacizumab (atezo-beva) or cabozantinib (atezo-cabo) has been granted approval for the treatment of advanced hepatocellular carcinoma (HCC). Given the current uncertainty among physicians and patients regarding the optimal choice between these two strategies, it becomes imperative to conduct a cost-effectiveness analysis to compare their relative benefits.

**Objective:**

Assessing the cost-effectiveness of atezo-beva compared to atezo-cabo in the treatment of advanced HCC.

**Methods:**

Both the network treatment comparison and cost-effectiveness analysis included patients from the IMbrave150 and the COSMIC-312 phase 3 randomized clinical trials. The network treatment comparison which included 761 patients was carried out, and a cost-effectiveness analysis that included 487 patients was conducted by developing a Markov model, both between February and November 2020. The robustness of the model was assessed via one-way and probabilistic sensitivity analyses. life-years, quality-adjusted life-years (QALYs), overall costs, and incremental cost-effectiveness ratios (ICERs) were measured.

**Results:**

Compared with the atezo-cabo group, atezo-beva group respectively increased 0.528 life years and 0.365 QALYs, with costs per patient increasing by $506,639, and the ICER was $1,388,052.054 per QALY. The one-way sensitivity analysis results indicated that the hazard ratios of OS and HR of PFS were the most sensitive factors in the model. In probabilistic sensitivity analysis, when the WTP threshold is $150,000 per QALY, the cost-effectiveness probabilities of the atezo-beva group and atezo-cabo group were 7.09% and 92.91% respectively.

**Conclusions:**

Findings from this cost-effectiveness analysis suggested that, compared to atezo-cabo, atezo-beva brought better OS benefits to advanced HCC patients, but also brought higher economic burden, considering the willingness-to-pay threshold was $150 000 per QALY, atezo-beva was not considered cost-effective.

## Introduction

As the third most significant contributor to cancer-related deaths worldwide, hepatocellular carcinoma (HCC) presents a formidable global healthcare challenge [1]. Although early detection of HCC enables treatment choices like surgical resection or liver transplantation, merely 30%−40% of HCC patients receive early diagnoses [2,3]. The majority of patients receive diagnoses at advanced stages, resulting in a grim prognosis [4]. A decade ago, the treatment options for advanced or unresectable HCC primarily involved multi-kinase inhibitors like Sorafenib, which managed to increase the median overall survival (OS) to 10.7 months [5]. Nevertheless, Sorafenib’s effectiveness is constrained, and it comes with a significant risk of drug-related adverse events (AEs), leading to suboptimal treatment outcomes [6,7].

Currently, several immunotherapy approaches are under evaluation for the treatment of HCC, and atezolizumab is a humanized monoclonal antibody that inhibits programmed cell death protein 1 (PD-1) [8]. Bevacizumab is a humanized monoclonal antibody that targets vascular endothelial growth factor (VEGF), thus inhibiting angiogenesis and tumor growth [8]. Cabozantinib is a tyrosine kinase inhibitor (TKI) with immunomodulatory properties [9]. Furthermore, anti-VEGF and TKI treatments can augment PD-1 inhibition, leading to a reduction in immunosuppression and the promotion of T-cell infiltration in tumors [10,11].

Two phase 3 clinical trials, namely IMbrave150 and COSMIC-312, have demonstrated that, when compared to standard treatment involving sorafenib, the combination therapies of atezolizumab plus bevacizumab (atezo-beva) yielded superior progression-free survival (PFS) and overall survival (OS) outcomes, atezolizumab with cabozantinib (atezo-cabo) just yielded PFS [8,9]. In the production and utilization of drugs, pricing based on the value of drugs to make the price of drugs cost-effective is a hot topic [12]. The rational pricing of drugs based on the value of drugs is a more reasonable pricing method, which can maximize the life span and quality of life of patients, and bring health benefits to patients and society [13]. Whether atezo-cabo can become another option needs to be explored the cost of atezo-beva is cost-effective also needs to be investigated.

To the best of our knowledge, there have been no published cost-effectiveness analyses comparing atezo-beva and atezo-cabo for the treatment of advanced or unresectable HCC. Both atezo-beva and atezo-cabo bring survival benefits to patients, but there is a lack of comparison of survival benefits and economic burden between them. Therefore, we performed a network meta-analysis to compare survival benefits and to provide important parameters for the cost-effectiveness analysis, this analysis assessed the cost-effectiveness of atezo-beva compared to atezo-cabo for the treatment of advanced or unresectable HCC patients from the perspective of U.S. healthcare payers.

## Methods

### Network treatment comparison

When the target treatments have not been studied head-to-head, network treatment comparison possibly provides more comprehensive evidences [14], combining direct within-trial evidence with indirect drug comparisons from other trials [15]. Without direct comparison of atezo-beva and atezo-cabo within-trial, a network treatment comparison was conducted on randomized controlled trials (RCTs) by systematically searching the Web of Science, PubMed and Embase from their inception until August 5th, 2023. This search was limited to studies in the English language. The search strategies were showed in S1 File. Details of the study selection are depicted in S1 Fig and the inclusion and exclusion criteria were showed in S2 File. The quality assessment of included studies was conducted using the Cochrane Risk of Bias tool [16]. This network treatment comparison was completed in R version 4.1.2 (R Project for Statistical) using the gemtc package to obtain hazard ratios (HR) for OS and PFS between atezo-beva and atezo-cabo. Due to the lack of data assessing heterogeneity between trials, a fixed-effects model was chosen for the analysis [17].

### Cost-effectiveness analysis

In the cost-effectiveness analysis, this study compared atezo-beva with atezo-cabo, all of which are approved immunotherapies for the treatment of advanced or unresectable HCC patients in the United States. Costs and benefits were discounted at an annual rate of 3% [18]. This study measured the life years, quality-adjusted life years (QALYs), total costs, and incremental cost-effectiveness ratios (ICERs) between treatments. The willingness-to-pay (WTP) for each QALY is $150,000. This study was conducted, and its findings were presented in adherence to the reporting guidelines established by the Consolidated Health Economic Evaluation Reporting Standards (CHEERS) [19]. This study did not employ individual patient-level data to inform the analysis. Consequently, it does not meet the criteria for human subject research and is not subject to review, exemption, or approval by an institutional review board or ethics committee, as outlined by the US Department of Health and Human Services (Table 1).

**Table 1:** Key Model Inputs.

Parameter	Expected value (range)	Distribution	References
Clinical input			
Survival model for atezo-cabo			
Weibull model for OS	Lambda = 0.01546928 gamma = 1.385069		Model fitting
Lognormal model for PFS	Mu = 1.908634 Sigma = 0.9985375		Model fitting
HR for OS	0.64 (0.42-0.97)	Log-normal	Network treatment comparison
HR for PFS	0.95 (0.61-1.46)	Log-normal	Network treatment comparison
Proportion of receiving subsequent active treatment			
Atezo-beva arm	0.21 (0.168-0.252)	Beta	[8]
Atezo-cabo arm	0.20 (0.16-0.24)	Beta	[9]
Atezo-beva arm：incidence of Grade ≥3 AEs			
Aspartate aminotransferase increased	0.07 (0.056-0.084)	Beta	[8]
Alanine aminotransferase increased	0.036 (0.029-0.043)	Beta	[8]
Hypertension	0.152 (0.122-0.182)	Beta	[8]
Atezo-cabo arm：incidence of grade ≥3 AEs			
Aspartate aminotransferase increased	0.09 (0.072-0.108)	Beta	[9]
Alanine aminotransferase increased	0.08 (0.064-0.096)	Beta	[9]
Hypertension	0.09 (0.072-0.108)	Beta	[9]
Utility input			
Utility of PFS	0.76 (0.61-0.91)	Beta	[20]
Utility of PD	0.68 (0.54-0.82)	Beta	[20]
Disutility due to AEs			
Grade 3 and higher	0.160 (0.110-0.204)	Beta	[20]
AEs cost, $/event			
Aspartate aminotransferase increased	59 (47.2-70.8)	Gamma	[21]
Alanine aminotransferase increased	59 (47.2-70.8)	Gamma	[21]
Hypertension	1,701 (1276-2127)	Gamma	[20]
Drug cost ($)			
Atezolizumab/10 mg	82.678 (66.142-99.214)	Gamma	[22]
Bevacizumab/10 mg	73.579 (58.863-88.295)	Gamma	[22]
Cabozantinib/mg	9.44 (7.552-11.328)	Gamma	[23]
Testing	797.33 (637.864-956.796)	Gamma	[24]
Subsequent active treatment per patient	108336 (81252-135420)	Gamma	[20]
Subsequent best supportive care per patient	37084 (27813-46355)	Gamma	[20]
Other			
Discount	0.03 (0.00-0.05)	Beta	[18]

Atezo-cabo, atezolizumab plus cabozantinib; OS, overall survival; PFS, progression-free survival; HR, hazard ratio; Atezo-beva, atezolizumab plus bevacizumab; AEs, adverse events; PD, progression-disease.

#### Population and interventions.

The patient group was derived from randomized clinical trials IMbrave150 and COSMIC-312 [8,9], and the baseline characteristics of the patients are outlined in S1 Table. The IMbrave150 trial commenced on March 15, 2018, and for this analysis, data up until August 29, 2019 were utilized. On the other hand, the COSMIC-312 trial began on April 12, 2019, and the analysis was based on data up until the cutoff date of March 8, 2021. Patients in the atezo-beva group and the atezo-cabo group received atezolizumab (1200 mg) in combination with bevacizumab (15 mg/kg body weight) or cabozantinib (40 mg orally once daily), administered intravenously every 3 weeks [8,9]. The average weight was 70 kg, used for calculating the bevacizumab dosage [17]. Subsequent treatment was administered to patients upon disease progression or occurrence of unacceptable adverse events [20,24,25].

#### Model structure.

**Fig 1:**
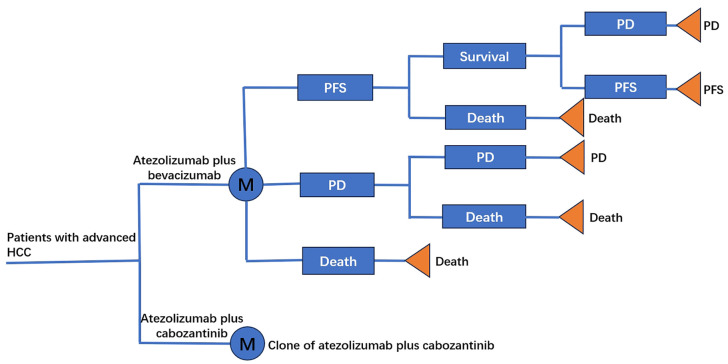
Markov model. M, Markov node; HCC, hepatocellular carcinoma; PFS, progression-free survival; PD, progression-disease.

A Markov model was constructed, comprising three separate and autonomous health states: PFS, progressive disease (PD), and Mortality (Death), as illustrated in Fig 1. All patients entered the Markov model with PFS state, the state would be changed after one cycle (3-weeks), patients would remain in PFS status unless PD or death, patients with PD state could remain in PD or change to death at the end of cycle [26]. According to the method proposed by Andrew et al [27], transition probabilities between these states were computed by leveraging the PFS and OS curves. The HRs of PFS and PD were used to get the transition probabilities of atezo-beva group. To capture all relevant fluctuations in costs and outcomes across the assessed options, the analysis was conducted over a lifetime horizon, extending until the demise of all patients within the model.

#### Effectiveness.

In the construction of the Markov model, visual data from the IMbrave150 and COSMIC-312 trials were obtained utilizing GetData Graph Digitizer, version 2.26 [28]. Time-to-event data were extracted in accordance with the methodology delineated in the study by Guyot et al [29]. These data were used to fit weibull, log-normal, log-logistic, exponential parametric survival functions. Subsequently, utilizing the Akaike Information Criterion (AIC), we selected the most appropriate survival function by identifying the model with the lowest AIC value (S2 Table). The weibull model was used for OS and the lognormal model was used for PFS. Subsequently, we derived the shape parameter (c) and the scale parameter (k) from this fitting process. Kaplan-Meier curves were then constructed using R software (http://www.rproject.org) [30].

Health utility values were allocated along a continuum from 0 (indicating death) to 1 (representing optimal health). Given the absence of health utility data for PFS and PD in the IMbrave150 and COSMIC-312 trials, we adopted health utility values from previously published literature [31]. Additionally, the disutility values linked to adverse events (AEs) were sourced from existing literature [8,9]. All utility and disutility values were used to achieve health effects.

#### Cost.

The analysis encompassed direct medical expenditures, encompassing expenses related to drug procurement, testing, subsequent active treatment, subsequent best supportive care, and management of adverse events. Drug costs were determined using the 2023 mean sale prices as outlined by the Centers for Medicare & Medicaid Services [22]. The costs for cabozantinib, testing, subsequent active treatment, subsequent best supportive care and management costs linked to adverse events were obtained from previously published literature [20,21,23,24]. The adverse events included aspartate aminotransferase increased, alanine aminotransferase increased and hypertension. The costs of subsequent active treatment and subsequent best supportive care were estimated from a cost-effectiveness analysis of second-line treatments of advanced HCC [20]. The cost of adverse effects was calculated in the first cycle, the cost of testing was calculated in each cycle before progression, and the cost after progression was composed of subsequent active treatment and subsequent best supportive care. Proportion of receiving subsequent active treatment and incidence of AEs were used to calculate the costs of subsequent active treatment and AEs. All costs were adjusted to 2023 US dollars using the Consumer Price Index.

#### Sensitivity analyses.

To assess the model’s robustness, we conducted both one-way sensitivity analyses and probabilistic sensitivity analyses. In a one-way sensitivity analysis, we derived variables from credible intervals or by assuming deviations of ±20% from the base-case values (Table 1). For the cost parameters, we opted for a gamma distribution, while for proportion, probability, and preference value parameters, we used a beta distribution. Additionally, for hazard ratios (HRs), we employed a log-normal distribution. In the probabilistic sensitivity analysis, we executed 10,000 Monte Carlo simulations, incorporating values randomly sampled from their respective statistical distributions.

## Result

### Network treatment comparison

Through database searching, 486 records were identified, and 2 phase 3 randomized clinical trials (IMbrave150 and COSMIC-312) involving 761 patients were included (S2 Fig). In the IMbrave150 trial, 329 patients were given atezo-beva treatment; in the COSMIC-312, 250 patients were given atezo-cabo treatment. The risk of bias is presented in S3 Fig. The network treatment comparison showed that, for the total population, the HR for PFS of atezo-beva compared to atezo-cabo was 0.95 (0.61, 1.46), and the HR for OS was 0.64 (95% CI, 0.42, 0.97).

### Cost-effectiveness analysis

#### Base-case analyses.

For the total population of patients, compared with the outcomes using atezo-cabo, atezo-beva group increased 0.528 life years and 0.365 QALYs, with an overall cost increasing by $506,639. (Table 2).

**Table 2:** Summary of cost and outcome results in the base-case analysis.

Factor	atezo-beva	atezo-cabo	Incremental
LYs	1.998	1.470	0.528
QALYs	1.428	1.063	0.365
Overall cost	1,427,598	920,959	506,639
ICER, $/LY	–	–	959,543.561
ICER, $/QALY	–	–	1,388,052.054

Atezo-cabo, atezolizumab plus cabozantinib; Atezo-beva, atezolizumab plus bevacizumab; LYs, life-years; QALYs, quality-adjusted life-years; ICER, incremental cost-effectiveness ratio.

#### Sensitivity analyses.

**Fig 2:**
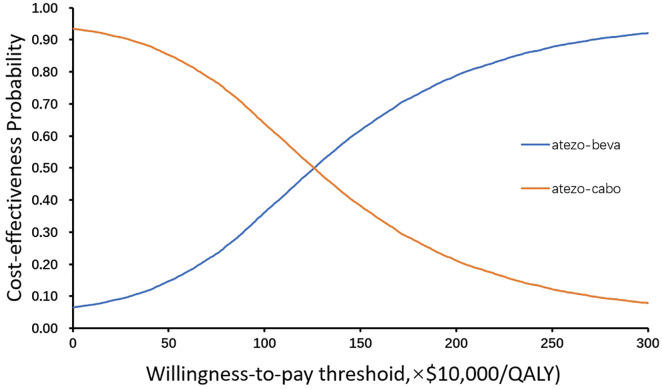
Cost-effectiveness acceptability curves. Atezo-beva, atezolizumab plus bevacizumab; Atezo-cabo, atezolizumab plus cabozantinib; QALY, quality-adjusted life-year.

For the atezo-beva vs atezo-cabo, we explored the association of key parameters with the ICER. The primary driver of the model outcomes was the HR of OS, followed by the HR of PFS and the utility of PD. The results of one-way sensitivity analyses showed that the results of the model did not significantly change when all parameters changed within a given range. We conducted probabilistic sensitivity analyses through 10,000 Monte Carlo simulations, the results were displayed in the scatterplot and the cost-effectiveness acceptability curves (Fig 2 and S5 Fig). When the WTP threshold was set at $150,000 per QALY, the atezo-beva group and the atezo-cabo group exhibited cost-effectiveness probabilities of 7.09% and 92.91%, respectively. At the WTP threshold of $1,255,850 per QALY, the cost-effectiveness probability of atezo-beva and atezo-cabo were equal, atezo-beva were cost-effective.

## Discussion

In the midst of escalating healthcare costs, there is an increasing emphasis on value-based oncology, with substantial interest directed towards immunotherapy approaches [32]. In this study, a network treatment comparison and a Markov model were employed as fundamental analyses, taking the perspective of U.S. payers into consideration. We evaluated the outcomes of two phase 3 randomized controlled trials, namely IMbrave150 and COSMIC-312, which collectively included 761 patients [8,9]. The results revealed that when compared to atezo-cabo, the HR for OS was 0.64 (95% CI, 0.42, 0.97) and the HR for PFS was 0.95 (95% CI, 0.61, 1.46) in the atezo-beva group. In the IMbrave150, compared to sorafenib, the HR for OS was 0.58 (95% CI, 0.42, 0.79) and the HR for PFS was 0.59(95% CI, 0.47, 0.76) in the atezo-beva group [8]. In the COSMIC-312, the HR for OS was 0.90 (95% CI, 0.69, 1.18) and the HR for PFS was 0.63(95% CI, 0.44,0.91) in the atezo-cabo group [9]. Intuitive analysis and comparison of HRs, our results were consistent with the two trials.

The incremental survival periods for the atezo-beva group compared to atezo-cabo group were 0.365 QALYs, with incremental costs per patient of $506,639. The calculated ICER were $1,388,052.054 per QALY. One-way sensitivity analysis shows that the HR of OS was the most sensitive factor affecting the ICER, followed by the HR of PFS and the utility PD. There was no significant difference in PFS between atezo-beva and atezo-cabo, but there was a significant difference in OS, the duration of PD would affect treatment costs and survival benefits. According to results from our comprehensive deterministic and probabilistic sensitivity analysis, the results of this model are robust. For the total population, at the WTP threshold of $150,000 per QALY, atezo-beva were not considered cost-effective, but atezo-beva were cost-effective at $1,255,850 per QALY. New drugs and treatments are often associated with high costs, and it is imperative to establish affordable prices for innovative therapies through cooperation between government and enterprises, government can provide funding and policies to support research and development, expand the coverage in medical insurance [33,34], and companies that can help patients through donation programs, etc.

Although some studies have evaluated the cost-effectiveness of atezo-beva compared with sorafenib [20,24,25,35–44], there is no study on atezo-cabo. The reason may be that atezo-cabo does not provide a good OS benefit, but we cannot neglect its advantage in PFS. In terms of high drug costs and research costs, as well as the interests of enterprises, phase III clinical trials comparing the efficacy of drugs from different pharmaceutical companies are currently difficult to achieve. As an effective means to deal with the lack of direct comparison results, network meta-analysis provides an excellent solution to compare the effectiveness of drugs from different pharmaceutical companies, and has been effectively used in the cost-effectiveness analysis of some drugs for the treatment of liver cancer [39,41,43,44].

The merits of this research warrant highlighting. Firstly, as far as we are aware, this study marks the inaugural assessment of the cost-effectiveness of atezolizumab in combination with bevacizumab compared to atezolizumab plus cabozantinib for advanced or unresectable HCC, drawing from the most recent RCTs available [45]. Secondly, our investigation employed a network meta-analysis methodology to conduct an indirect comparison of checkpoint inhibitors. Furthermore, we established a Markov model that incorporates variables to comprehensively explore the cost-effectiveness of immunotherapies [31].

Nonetheless, it is important to acknowledge certain limitations of this analysis. Firstly, we had to make assumptions regarding health outcomes that extend beyond the follow-up periods of the IMbrave150 and COSMIC-312 trials by extrapolating data based on the published Kaplan-Meier overall survival (OS) and progression-free survival (PFS) data, this could introduce a degree of uncertainty into the model outputs. Expanded experimental data with more patients and a large amount of real-world data might reduce uncertainty and promote model results to be more realistic. However, it’s worth noting that this limitation may not have a significant impact, as indicated by the results of our sensitivity analysis, which suggested that this finding is generally robust [46]. Secondly, it’s important to recognize that both trials are Phase 3 randomized controlled trials (RCTs), and the parameters within the model rely on the outcomes observed in these two trials [47]. Hence, it’s essential to acknowledge that the cost and effectiveness outcomes could potentially be influenced by any biases inherent to the trial. For instance, the trial participants tended to be in better overall health compared to the broader population of patients with advanced or unresectable HCC. Additionally, clinical trial participants typically exhibit higher treatment adherence when compared to patients in real-world practice [48,49]. In the actual clinical practice, and the cost and curative effect of treatment will be different from clinical trials, the choice of treatment scheme needs more comprehensive consideration of doctors and patients, an excellent treatment plan should be an individualized plan based on comprehensive consideration of the patient’s condition, compliance with treatment guidelines, respect for clinical trial results, and consideration of the patient’s treatment intention and economic status.

## Conclusions

Findings from this cost-effectiveness analysis suggested that, compared to atezo-cabo, atezo-beva brought better OS benefits to advanced HCC patients, but also brought higher economic burden, considering the willingness-to-pay threshold was $150,000 per QALY, atezo-beva was not considered cost-effective.

## Supporting information

S1 Fig Flowchart of study selection. (TIF)

S2 Fig Network graph of treatment comparison. Nodes represent competing treatments. A, Sorafenib; B, atezolizumab plus bevacizumab; C, atezolizumab plus cabozantinib.
(TIF)

S3 Fig Risk of bias summary. Richard S in 2020 represents the IMbrave150 clinical trial; Robin K in 2022 represents the COSMIC-312 clinical trial.
(TIF)

S4 Fig Tornado diagram of one-way sensitivity analyses. HR, hazard ratio; OS, overall survival; PFS, progression-free survival; PD, progression-disease; AB, atezolizumab plus bevacizumab; BSC, best supportive care; SAT, subsequent active treatment; AC, atezolizumab plus cabozantinib.
(TIF)

S5 Fig Scatter plot of probabilistic sensitivity analyses. WTP, willingness to pay.
(TIF)

S1 Table Patient baseline characteristics. ECOG,Eastern Cooperative Oncology Group, scores range from 0 to 5, with higher numbers reflecting greater disability; BCLC,Barcelona Clinic liver cancer stage; Atezo-beva, atezolizumab plus bevacizumab; Atezo-cabo, atezolizumab plus cabozantinib.
(DOCX)

S2 Table Distribution, parameter values, and AIC in Atezo-cabo group. AIC, Akaike’s information criterion; Atezo-cabo, atezolizumab plus cabozantinib; OS, overall survival; PFS, progression-free survival.
(DOCX)

S1 File Search Strategies. (DOCX)

S2 File Inclusion and exclusion criteria. (DOCX)
