# Supplementary figures and images for: Comparison of Atezolizumab plus Aevacizumab and Atezolizumab plus Aabozantinib for advanced hepatocellular carcinoma: A cost-effectiveness analysis
Source: PLoS One. 2025 Dec 3;20(12):e0337606. doi: 10.1371/journal.pone.0337606 (PMC12674557; doi:10.1371/journal.pone.0337606)

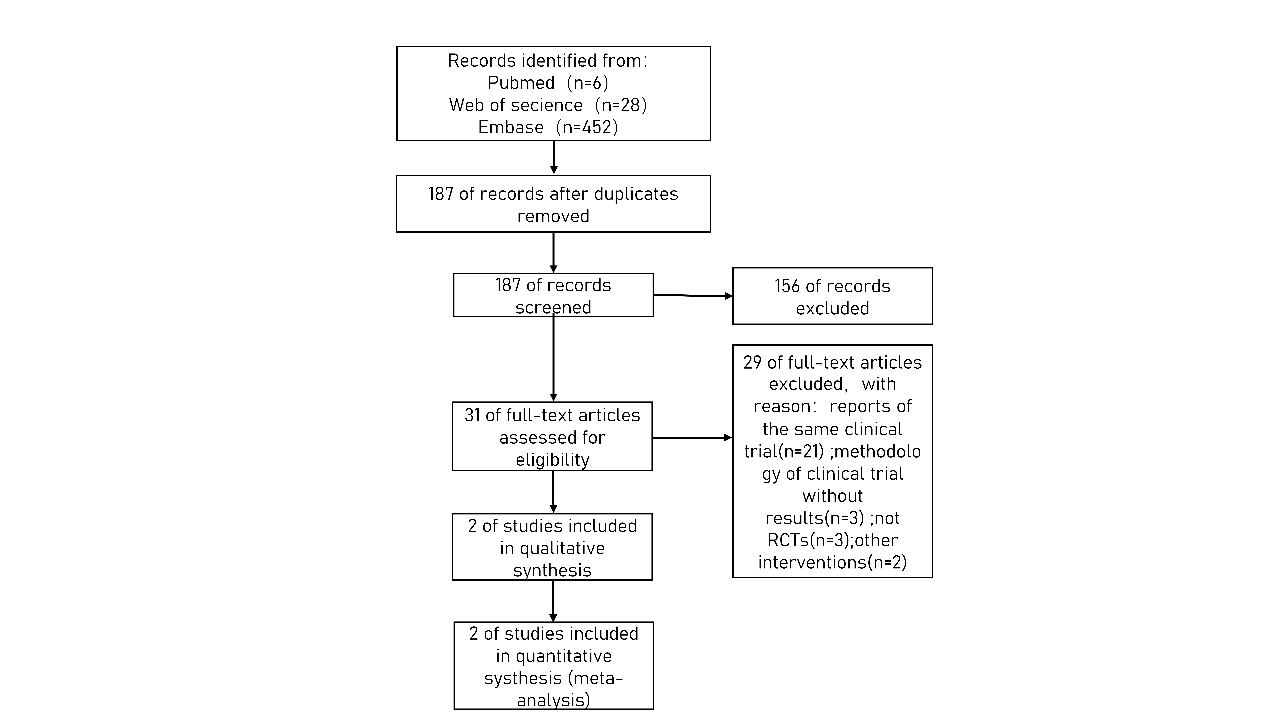

Supplement: S1 Fig — (TIF) [file pone.0337606.s001.tif]

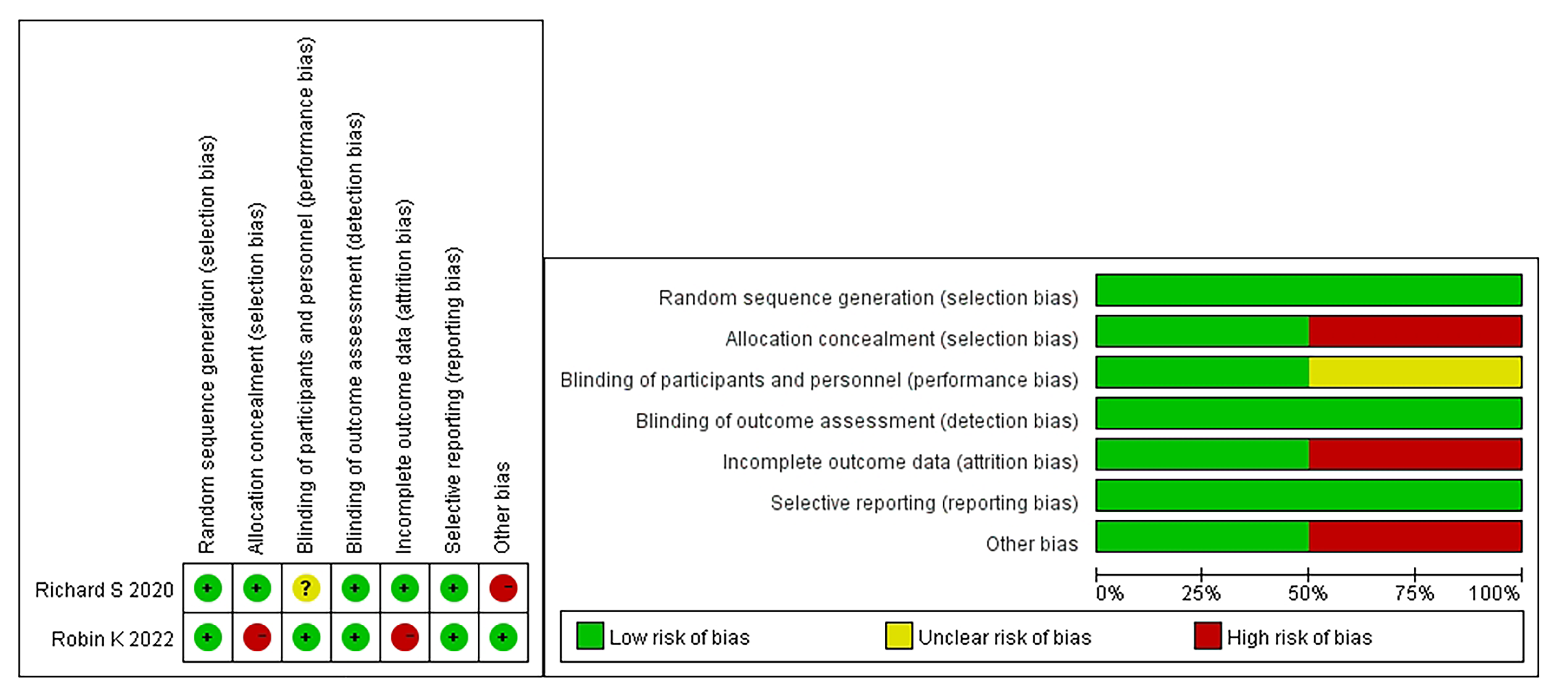

Supplement: S3 Fig — Richard S in 2020 represents the IMbrave150 clinical trial; Robin K in 2022 represents the COSMIC-312 clinical trial. (TIF) [file pone.0337606.s003.tif]

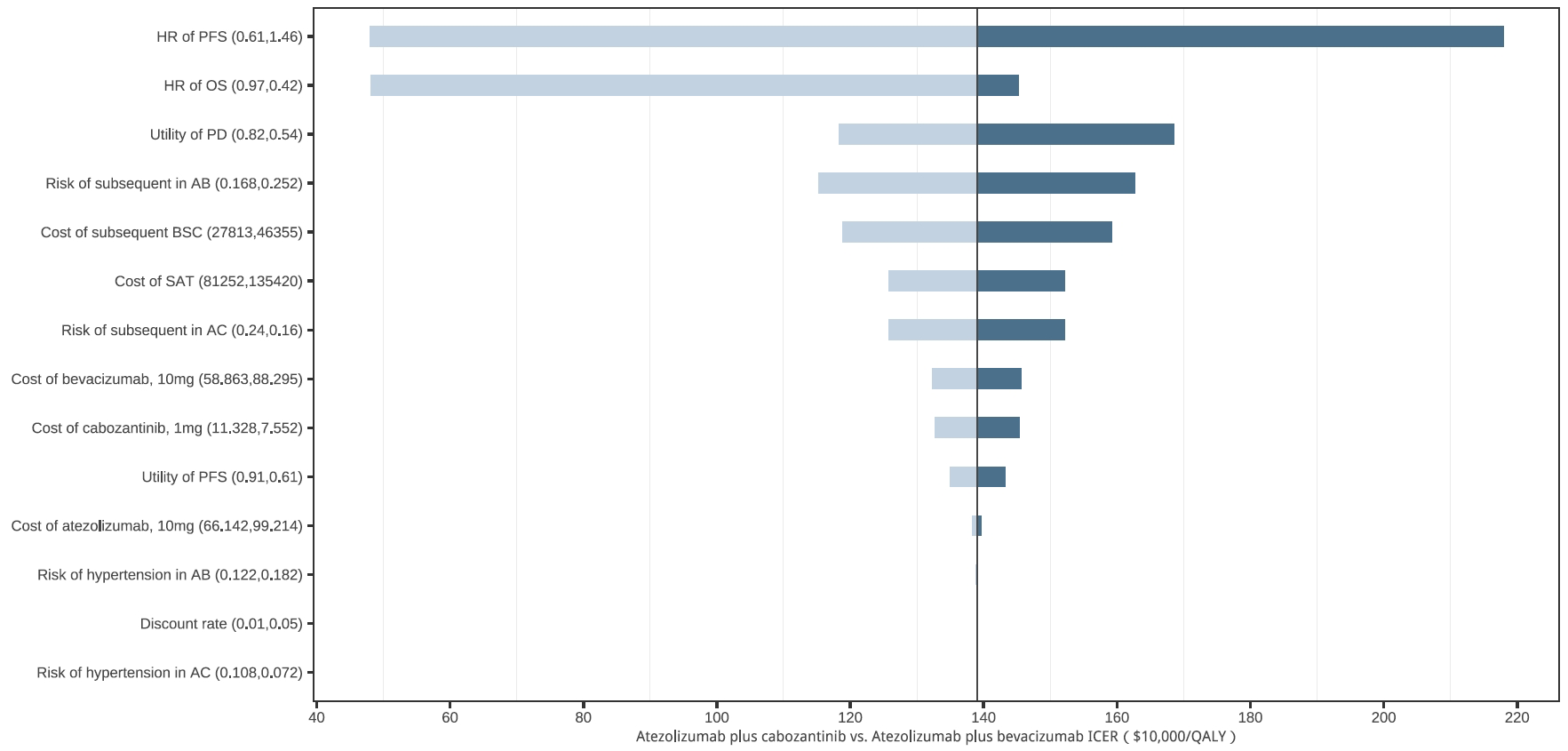

Supplement: S4 Fig — HR, hazard ratio; OS, overall survival; PFS, progression-free survival; PD, progression-disease; AB, atezolizumab plus bevacizumab; BSC, best supportive care; SAT, subsequent active treatment; AC, atezolizumab plus cabozantinib. (TIF) [file pone.0337606.s004.tif]

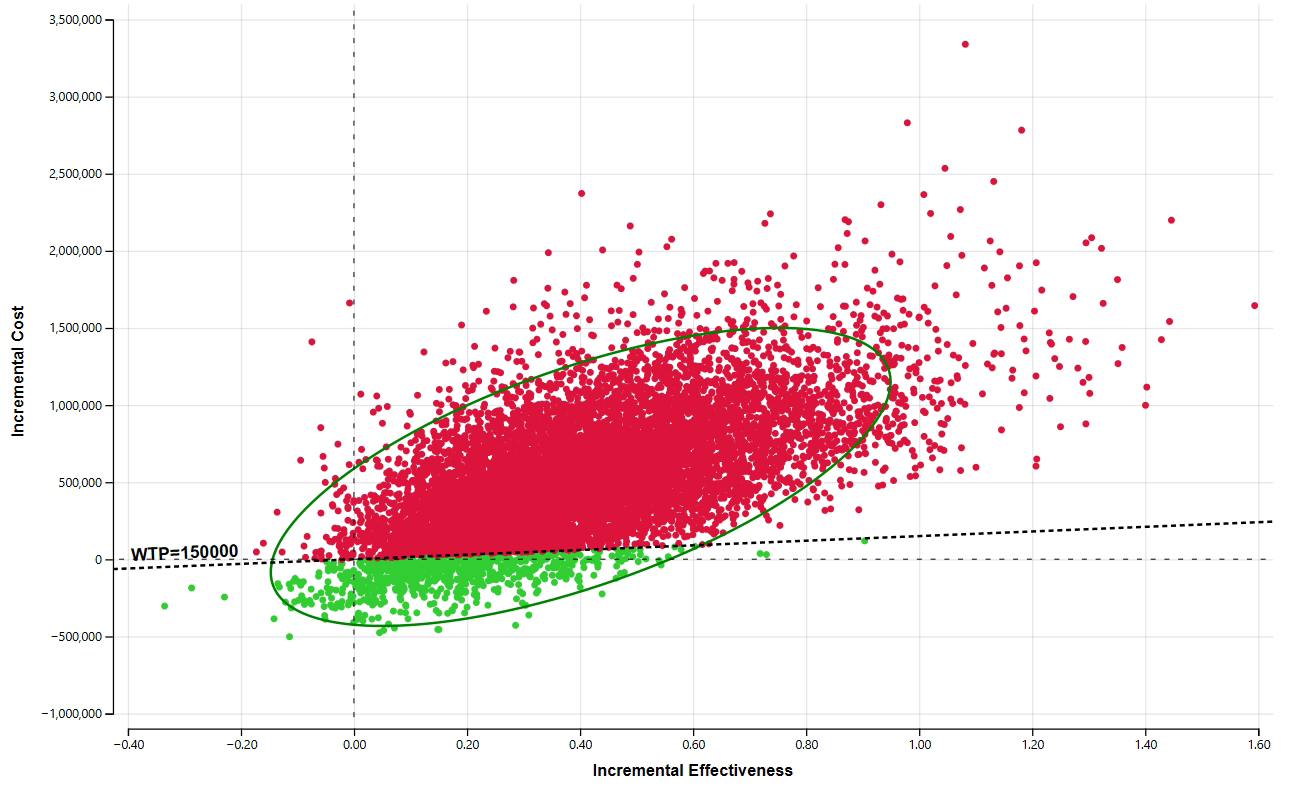

Supplement: S5 Fig — WTP, willingness to pay. (TIF) [file pone.0337606.s005.tif]
