# Supplementary material for: Comparison of Atezolizumab plus Aevacizumab and Atezolizumab plus Aabozantinib for advanced hepatocellular carcinoma: A cost-effectiveness analysis
Source: PLoS One. 2025 Dec 3;20(12):e0337606. doi: 10.1371/journal.pone.0337606 (PMC12674557; doi:10.1371/journal.pone.0337606)
Supplement: S1 Table — ECOG,Eastern Cooperative Oncology Group, scores range from 0 to 5, with higher numbers reflecting greater disability; BCLC,Barcelona Clinic liver cancer stage; Atezo-beva, atezolizumab plus bevacizumab; Atezo-cabo, atezolizumab plus cabozantinib. (DOCX) [file pone.0337606.s006.docx]

S1 Table. Patient baseline characteristics.

| Characteristic | Atezo-beva group | Atezo-cabo group |
| --- | --- | --- |
| Median Age (range)—years | 64 (56–71) | 65 (58–70) |
| Male sex | 277 (82%) | 214 (86%) |
| ECOG performance status score |  |  |
| 0 | 209 (62%) | 162 (65%) |
| 1 | 127 (38%) | 87 (35%) |
| Aetiology of disease |  |  |
| Hepatitis B virus | 164 (49%) | 74 (30%) |
| Hepatitis C virus | 72 (21%) | 71 (28%) |
| BCLC stage |  |  |
| B (intermediate) | 52 (15%) | 83 (33%) |
| C (advanced) | 276 (82%) | 167 (67%) |
| Race |  |  |
| Asian | 133 (40%) | 67 (27%) |
| Other | 203 (60%) | 136 (54%) |
| Subsequent therapy | 69 (20.5%) | 87 (20%) |
| ECOG,Eastern Cooperative Oncology Group, scores range from 0 to 5, with higher numbers reflecting greater disability; BCLC,Barcelona Clinic liver cancer stage; Atezo-beva, atezolizumab plus bevacizumab; Atezo-cabo, atezolizumab plus cabozantinib. | | |
