# Supplementary material for: Comparison of Atezolizumab plus Aevacizumab and Atezolizumab plus Aabozantinib for advanced hepatocellular carcinoma: A cost-effectiveness analysis
Source: PLoS One. 2025 Dec 3;20(12):e0337606. doi: 10.1371/journal.pone.0337606 (PMC12674557; doi:10.1371/journal.pone.0337606)
Supplement: S2 Table — AIC, Akaike’s information criterion; Atezo-cabo, atezolizumab plus cabozantinib; OS, overall survival; PFS, progression-free survival. (DOCX) [file pone.0337606.s007.docx]

S2 Table. Distribution, parameter values, and AIC in Atezo-cabo group.

| Model | a | **b** | **AIC** |
| --- | --- | --- | --- |
| OS curve | | | |
| Exponential | 0.04075211 |  | 1581.293 |
| Weibull | 0.01546928 | 1.385069 | 1561.638 |
| Log-normal | 2.811808 | 1.174498 | 1570.198 |
| Log-logistic | 16.00776 | 1.585295 | 1564.372 |
| PFS curve | | | |
| Exponential | 0.09894378 |  | 1135.116 |
| Weibull | 0.05478483 | 1.266116 | 1124.471 |
| Log-normal | 1.908634 | 0.9985375 | 1109.307 |
| Log-logistic | 6.786011 | 1.690095 | 1115.449 |
| AIC, Akaike’s information criterion; Atezo-cabo, atezolizumab plus cabozantinib; OS, overall survival; PFS, progression-free survival. | | | |
