# Supplementary material for: Comparison of Atezolizumab plus Aevacizumab and Atezolizumab plus Aabozantinib for advanced hepatocellular carcinoma: A cost-effectiveness analysis
Source: PLoS One. 2025 Dec 3;20(12):e0337606. doi: 10.1371/journal.pone.0337606 (PMC12674557; doi:10.1371/journal.pone.0337606)
Supplement: S1 File — (DOCX) [file pone.0337606.s008.docx]

eMethods 1. Search Strategies.

Web of Science: ((TI=(atezolizumab)) AND TS=(hepatocellular carcinoma)) AND TI=(phase III or phase 3)

PubMed: (atezolizumab[Title]) AND ((hepatocellular carcinoma[Title/Abstract]) AND ((phase III[Title]) OR (phase 3[Title])))

Embase: atezolizumab AND hepatocellular AND carcinoma AND phase AND (iii OR 3)
