# Supplementary material for: Comparison of Atezolizumab plus Aevacizumab and Atezolizumab plus Aabozantinib for advanced hepatocellular carcinoma: A cost-effectiveness analysis
Source: PLoS One. 2025 Dec 3;20(12):e0337606. doi: 10.1371/journal.pone.0337606 (PMC12674557; doi:10.1371/journal.pone.0337606)
Supplement: S2 File — (DOCX) [file pone.0337606.s009.docx]

eMethods 2. Inclusion and exclusion criteria.

Inclusion criteria:

A. Patients were aged 18 years or older with pathological diagnosis of hepatocellular carcinoma;

B. Treatment in the trial group was atezolizumab plus bevacizumab or atezolizumab plus cabozantinib;

C. The study type was a phase III randomized controlled trial;

D. Results for overall survival and progression-free survival are reported.

Exclusion criteria:

A. Non-english literature, conference abstracts, and duplicate publications;

B. The study was not a phase III clinical trial or the treatment was not first-line therapy;

C. Studies with incomplete data information or where the required data could not be obtained.
